# INVESTIGATION OF INDEPENDENCE IN THE TASKS INVOLVING THE USE OF PUBLIC TRANSPORTATION IN PATIENTS WITH SUBACUTE STROKE

**DOI:** 10.2340/jrm.v57.42025

**Published:** 2025-03-12

**Authors:** Shin KITAMURA, Yohei OTAKA, Kazuki USHIZAWA, Seigo INOUE, Sachiko SAKATA, Kunitsugu KONDO

**Affiliations:** 1 Department of Rehabilitation Medicine, Tokyo Bay Rehabilitation Hospital, Chiba; 2 Faculty of Rehabilitation, School of Health Sciences, Fujita Health University, Aichi; 3 Department of Rehabilitation Medicine, School of Medicine, Fujita Health University, Aichi, Japan

**Keywords:** activities of daily living, cerebrovascular disorders, task performance and analysis

## Abstract

**Objective:**

To clarify the percentage of stroke patients who are independent in performing tasks involved in public transportation use and problems faced while doing so.

**Design:**

Single-institution retrospective study.

**Patients:**

A total of 237 post-stroke patients utilized public transportation during their hospitalization in subacute rehabilitation wards.

**Methods:**

Participants’ actual performance in 14 tasks involving public transportation use was assessed using the Public Transportation Use Assessment Form. For each task, the percentage of participants who could perform the task independently was calculated and identified performance problems were categorized.

**Results:**

The task with the lowest percentage of independent participants was “Walking in crowds”, with 146 of 236 (61.9%) participants performing this independently, followed by “Selecting departure time and platform” (149 of 229, 65.1%), and “Getting on and off trains” (162 of 230, 70.4%). Problems faced when “Walking in crowds” included the “Risk of colliding with others” (*n* = 34), “Stopping abruptly” (*n* = 16), “Lack of attention to surroundings” (*n* = 14), and “Unable to walk with the flow of people” (*n* = 11).

**Conclusion:**

A significant number of patients could not perform tasks independently and faced various performance problems. These issues should be addressed during rehabilitation to enable patients to use public transportation.

## LAY ABSTRACT

For stroke survivors, public transportation is the primary transportation mode for social participation, and rehabilitation often includes practising before resuming its use. Using public transportation includes many tasks requiring different movements and procedures, each with varying difficulty for independence. In this study, we examined stroke patients’ independence in performing 14 tasks required to use public transportation and identified each task’s difficulty. The results showed that the task with the fewest independent participants was “Walking in crowds” (61.9% performed independently), followed by “Selecting departure time and platform” (65.1%), and “Getting on and off trains” (70.4%). While some problems common to performing each task were observed, such as “Losing balance”, many were specific to each task. Understanding these challenges can contribute to developing focused practice plans for stroke patients, thus ensuring they can resume using public transportation.

Social participation is an important factor influencing the quality of life of individuals (1, 2), making it important to prioritize its promotion in stroke rehabilitation (3). When post-stroke patients begin to reintegrate into society, they frequently use public transportation as physical and cognitive impairments preclude them from driving vehicles owing to the increased risk of traffic accidents (4, 5). However, the use of public transportation presents challenges for post-stroke patients (4, 6–9), with some being unable to use trains and buses (4, 10–13), limiting their social participation (13–17). Therefore, rehabilitation interventions to help individuals resume using public transportation post-stroke are important for promoting their social participation (18, 19).

Using public transportation involves multiple tasks. For example, riding trains requires walking outdoors, passing through ticket gates, waiting at the appropriate platform, and getting on and off the train. Previous studies have identified 14 tasks required for post-stroke patients to use public transportation, and a Public Transportation Use Assessment Form (PTAF) was developed to assess the independence level regarding each task and the problems faced when performing these tasks (20, 21). The tasks pertaining to using public transportation entail different movements and procedures; thus, variations exist in task difficulty and the problems faced when performing them.

After a stroke, rehabilitation sometimes involves patients practising using public transportation (22, 23); however, constant practice can be difficult owing to various barriers, including ensuring patients’ safety, high costs due to personnel and time needs, and a lack of appropriate social services (e.g., medical insurance) (24). Therefore, few opportunities exist for patients to use public transportation in rehabilitation. Knowing which tasks stroke patients tend to be unable to perform independently and the problems faced in performing them could assist with efforts in practice that are focused on tasks that are expected to be challenging. One study used a questionnaire to examine difficulties across six aspects of public transportation use (i.e., transferring to and from, getting on and off, using the train or ferry facilities, orientation, booking and payment, and fear) and found that 3–16% of respondents reported difficulties post-stroke (8). Other studies have conducted interviews to clarify the range of difficulties experienced by individuals post-stroke when using public transportation, such as the cognitive load due to time pressures, the number of steps involved, and the physical demands of getting on and off a bus (6, 9, 25). However, the specific tasks involved in using public transportation have not been comprehensively and objectively evaluated, and the proportion of independent patients and problems encountered when performing these tasks remain unclear. The present study aimed to determine the percentage of stroke patients who can independently perform the tasks involved in public transportation use and the problems faced in performing these tasks.

## METHODS

### Study design and setting

This study retrospectively analysed the records of public transportation use practices at the Kaifukuki Rehabilitation Ward of Tokyo Bay Rehabilitation Hospital, a 160-bed facility in Japan, between October 2017 and January 2020. These wards provide subacute intensive rehabilitation covered by the medical insurance system, admitting patients with stroke within two months of symptom onset and allowing a maximum stay of six months (26).

In this hospital, patients could practise public transportation use according to their individual needs. The practice was conducted in the cities of Narashino (population of 172,000 in 2017) and Funabashi (population of 632,000 in 2017) in northwestern Chiba Prefecture (27), which is adjacent to Tokyo. From these cities, it takes 30–40 min to reach Tokyo by train. Both cities had relatively high train and bus usage, accounting for 30% of all transportation use (28). The daily number of passengers at each station used in practice ranged from 11,000–137,000 (29, 30).

All patient data used in this study, including the records of public transportation use practices, were extracted from the hospital’s medical records. Prior to the analysis, all data were anonymized by replacing the patient’s name with an identification code. The protocol for the present study was approved by the appropriate ethics committee. The requirements for informed consent were waived because of the retrospective study design.

### Participants

Among the 803 post-stroke patients admitted during the study period, 237 patients who used public transportation were enrolled in the present study. In the Tokyo Bay Rehabilitation Hospital, patients who met the following criteria were considered to practise using public transportation: (*i*) they were expected to use public transportation after hospital discharge, (*ii*) they or their family hoped to practise using public transportation, and (*iii*) their physical and occupational therapist and physician determined that such practice was necessary during their hospitalization. We gathered data from the hospital’s database to determine the participants’ clinical and demographic characteristics. The data included patient evaluations on the Mini-Mental State Examination-Japanese (MMSE-J) (31, 32), Stroke Impairment Assessment Set (SIAS) motor items and higher cortical function items (33, 34), and the Functional Independence Measure (FIM) (35, 36) during the practice period. The MMSE-J is an 11-item questionnaire that assesses cognitive function. Scores range from 0 to 30, with higher scores indicating better cognitive function. The SIAS motor items assess motor function on the hemiparetic side and consist of 5 items, with 6 grades from 0 (total paralysis) to 5 (normal) for each item. The SIAS higher cortical function items consist of the Visuospatial Perception item, which assesses unilateral spatial neglect, and the Speech item, which assesses aphasia. Each item is scored from 0 (severe) to 3 (no symptoms). The FIM assesses patients’ independence in activities of daily living, and consists of 13 motor and 5 cognitive items, with each item scored on a 7-point scale from 1 (total assistance) to 7 (complete independence); the motor score is the sum of the motor items (13–91 points) and the cognitive score is the sum of the cognitive items (5–35 points). The MMSE-J, SIAS, and FIM scores were assessed by an occupational therapist, physical therapist, and nurse, respectively.

### Practice of public transportation use

Each participant practised using public transportation accompanied by their physical therapist (PT) and occupational therapist (OT). The PTs and OTs assessed the participants’ performance during these sessions using the PTAF. The PTAF is a 15-item tool developed to evaluate post-stroke patients’ actual performance of individual tasks involved in public transportation use (Fig. S1) (20). It includes the item “other problems observed”, and the remaining 14 items are classified into 4 categories (i.e., Plans for going out, Mobility, Using trains, and Using buses). These categories assess patients’ independence in performing tasks related to public transportation usage. Each task is scored on a 4-point scale: 3=independent (i.e., the patient can complete the task without any assistance from a therapist), 2 = requiring supervision or verbal assistance (i.e., the patient can complete the task with the therapist’s supervision or verbal assistance), 1 = requiring physical assistance (i.e., the patient requires a therapist’s physical assistance to perform the task or requires the therapist to manipulate equipment, such as a ticket vending machine); and N = not applicable (i.e., patient does not need to perform the task; for example, if a patient uses only trains, the items in the category “Using buses” are not evaluated). If the patient could not perform the task independently (i.e., scores of 1 or 2), the specific difficulties in performing the task were recorded in the “Problems” section next to each item, which provided the opportunity for a free-response description. The PTs and OTs used the PTAF in their daily clinical practice and were familiar with using this tool for assessment. The PTAF has been confirmed to have sufficient reliability and validity (19).

The type of transportation used for practice was selected from 1 of 3 courses—Train only (Course 1), Bus only (Course 2), or Both Train and Bus (Course 3; Fig. S2). The course that was practised was selected based on discussions between the participant, their family members, physician, PT, and OT, considering the participant’s needs after hospital discharge. Before the practice day, participants were informed of the course and practice time by their therapists. The accompanying PTs and OTs provided physical or verbal assistance only when requested by the participant or when the therapists deemed it necessary for safety (e.g., fall prevention). The PTs and OTs assessed the patient’s performance through observation and, if necessary, by communicating with the patient, in order to identify patients’ perspectives, such as patients’ fatigue, pain, and difficulty in performing the task. In the “problems” section, behaviours were recorded when patients performed differently from the movement that the patients had practised as safe with their PTs and OTs during routine rehabilitation, and the PTs and OTs determined whether this behaviour interfered with the safe performance of each task. The PTs and OTs discussed and completed the PTAF immediately after the practice, resulting in a single assessment for each participant.

### Data analysis

We examined the percentage of participants who could independently perform the 14 tasks and 4 categories of the PTAF, as well as the 3 courses used in practice. First, we calculated the percentage of participants judged to be independent for all items except for the item “other problems observed”. Next, we calculated the percentage of participants judged to be independent in all included items for all categories and courses. Each course included a different number of items. Course 1 included 10 items across the categories Plans for going out, Mobility, and Using trains; Course 2 included 9 items across the categories Plans for going out, Mobility, and Using buses; Course 3 included 14 items across all 4 categories. Participants who had a score of “N, not applicable” in a task were excluded from the analyses. Participants with missing values were excluded in each analysis.

We extracted and classified the descriptive data in the Problems section of each PTAF item according to similarity in semantic content to determine the problems participants encountered while performing tasks. If a single description contained multiple issues, it was assigned multiple classifications (i.e., one descriptive response could be included in more than one classification). All descriptive data were classified and classifications consisting of more than one descriptive response were included in the analysis. We labelled each classification to reflect the data it encompassed. To determine the frequency of problems in performing each task, we counted the number of participants corresponding to data in each category (i.e., patients who were assessed by their therapists as having a problem in performing the task). The classification process was performed collaboratively by the first and third authors, after which the second author reviewed the results. If the second author deemed revisions necessary, the first and third authors reclassified the data. This iterative process continued until a consensus was reached.

## RESULTS

Participants’ characteristics are presented in Table I. Participants exhibited relatively high scores on the MMSE-J, SIAS items, and the FIM, indicating that many of them had mild impairments post-stroke. In all, 20 participants (8.4%) used Course 1 (Train only), 7 (2.9%) used Course 2 (Bus only), and 210 (88.6%) used Course 3 (Train and bus).

**Table I:** Patient characteristics (*n* = 237)

Sex: male/female	74/163
Age in years (mean [SD])	63.4 (13.9)
Type of stroke, *n*	93/121/23
Haemorrhage/infarction/subarachnoid haemorrhage	
Side of hemiparesis, right/left/none	127/100/10
Days after stroke onset, mean (SD)	92.8 (42.7)
Days after admission to rehabilitation hospital, mean (SD)	62.7 (39.6)
MMSE-J, median (IQR)	29 (3)*
SIAS: median (IQR)	
Knee–mouth	4 (1)
Finger–function	5 (1)
Hip–flexion	5 (1)
Knee–extension	5 (1)
Foot–pat	5 (1)
Visuospatial perception	3 (0)^†^
Speech	3 (1)^‡^
FIM, median (IQR)	
Motor score	86 (8)
Cognitive score	32 (6)
Total score	117 (12)

FIM: Functional Independence Measure; IQR: interquartile range; MMSE-J: Mini-Mental State Examination-Japanese; SIAS: Stroke Impairment Assessment Set, Missing data:

* = 15,

† = 2,

‡ = 11.

Table II presents the percentage of participants who could independently perform the tasks in each category and course. In Course 1, of the 13 participants who tried to perform all included tasks (i.e., the participants from the 20 participants who used Course 1, excluding the 7 participants who included tasks that were “N”), 2 (15.4%) performed all tasks independently. In Course 2, 2 (33.3%) of 6 participants could perform all tasks, and in Course 3, 75 (41.9%) of 179 participants could perform all tasks. Across all courses, a higher percentage of participants could independently perform the items in the “Plans for going out” category. The other categories did not significantly differ in the percentage of participants who could perform the tasks independently.

**Table II:** Percentage of participants who completed the task comprising each category and course

Course	Overall course	Categories			
		Plans for going out	Mobility	Using trains	Using buses
Course 1. Trains (*n =* 20)	2/13 (15.4)	14/20 (70.0)	4/15 (26.7)	4/15 (26.7)	-
Course 2. Buses (*n =* 7)	2/6 (33.3)	6/7 (85.7)	2/6 (28.6)	-	2/7 (28.6)
Course 3. Trains and buses (*n =* 210)	75/179 (41.9))*	160/208 (76.9)	118/208 (56.7)	99/186 (53.2)	113/198 (57.1))*

Number of missing values: *n*

* = 4. In each cell, the number to the left indicates the number of participants who were able to perform all tasks independently and the number to the right indicates the number of participants who practised all tasks included in the category or course. The numbers in parentheses indicate the percentage of participants who were able to perform all tasks in each course or category.

**Fig. 1:**
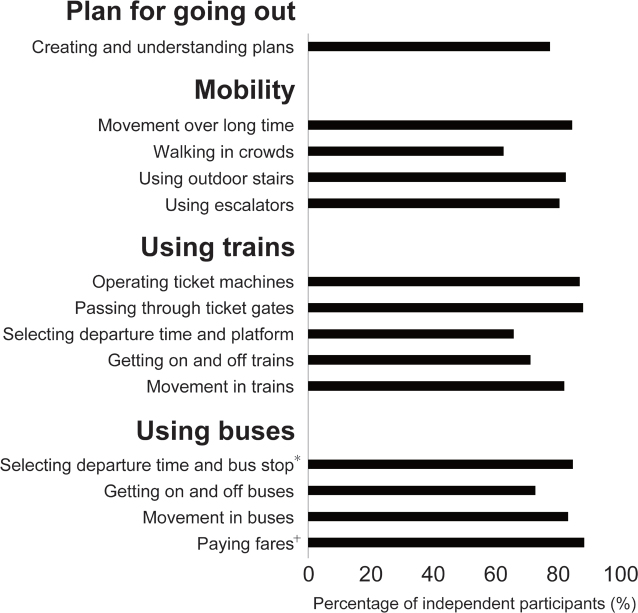
Percentage of participants who could independently perform each task involved in using public transport. Number of missing values: *n = 2, ^†^*n* = 3. **Bold text** indicates category names that include individual tasks. The percentage of participants judged to be independent after excluding those who scored “N” in each task involved in public transport use.

Fig. 1 shows the percentage of participants who could perform the 14 tasks independently. The task with the lowest percentage of independent participants was “Walking in crowds”, with 146 of 236 (61.9%) participants performing it independently, followed by “Selecting departure time and platform” (149 of 229, 65.1%), “Getting on and off trains” (162 of 230, 70.4%), and “Getting on and off buses” (156 of 217, 71.9%). Problems in performing each task are presented in Table III. Although some common problems were identified such as balance problems, improper procedures, and fatigue, many were specific to each task. “Walking in crowds” included the problems “Risk of colliding with others” (*n* = 34), “Stopping abruptly” (*n* = 16), “Lack of attention to surroundings” (*n* = 14), and “Unable to walk with the flow of people” (*n* = 11).

**Table III:** Problems performing each task independently

Problems in performing tasks	Details	n
**Creating and understanding plans**		**208**

Unable to understand plans	They could not follow the plan while understanding routes, times, and fees They faced difficulties in utilizing compensatory strategies such as memos	14
Unable to create plans	They could not develop a plan independently and needed help from others They could not utilize the internet to access information needed for planning	8
Confusion due to unfamiliar environment	They were unfamiliar with courses that they did not normally use and were confused regarding executing their plan	7

**Movement over long time**		**210**

Unstable gait	They stumbled and exhibited difficulty swinging their legs forward as they walked longer distances, and their postural control became unstable	21
Feeling fatigued	They became fatigued and, in some cases, needed to rest	16
Experiencing pain	They began to feel pain in their lower back and knees	3
Changes in vital signs	They were short of breath and their blood oxygen saturation decreased	2
Decrease in walking speed	Their walking speed decreased	2

**Walking in crowds**		**209**

Risk of colliding with others	They collided or nearly collided with people or objects around them	34
Stopping abruptly	They stopped abruptly in a crowded place, disrupting the flow of people around them	16
Lack of attention to surroundings	They could not walk while paying attention to their surroundings	14
Unable to walk with the flow of people	They could not walk with the flow of people due to slow walking speed	11

**Using outdoor stairs**		**205**

Losing balance	They lost their balance, tripped, or failed to shift their centre of gravity	17
Improper movement procedures	In their haste to keep up with the flow of people, they made mistakes in some procedures, such as the order of steps and whether to use handrails, and their movements became chaotic	9
Going up/down in improper position	Their positioning when going up and down (right or left side of the stairs) was opposite to the flow of people, interfering with others’ movements	4
Slow speed while going up/down stairs	Their speed of going up and down stairs was slow, and they could not move with the flow of people	4
Getting fatigued	They felt exhausted when going up large flights of stairs	3

**Using escalators**		**207**

Losing balance	They lost their balance when getting on and off escalators	9
Lack of attention to surroundings	They found it difficult to pay attention to their surroundings and obstructed the movement of others	9
Unable to get on/off with the motion of the escalator	They could not move their feet at the right time to match the motion of the escalators and stopped or slowed down when getting on and off escalators	8
Unable to get on/off with the flow of people	They could not get on/off with the flow of people and stopped or slowed down when getting on and off the escalator	6
Using escalators in dangerous positions	They used the escalator by riding in a dangerous position, such as riding with their feet sticking out of the steps	6
Improper movement procedures	Despite the need for support, they got on/off escalators without gripping the handrail	4

**Operating ticket machines**		**178**

Not understanding operating procedures	They could not understand procedures such as adding money to their IC cards or purchasing tickets	9
Not understanding fares	They could not recognize and calculate the required amount of money when checking the fare list	5
Difficulties taking out cash/prepaid IC cards	They took a long time or needed assistance to take out their wallets (cash) or cards	3

**Passing through ticket gates**		**203**

Difficulty taking out prepaid IC cards/smartphone	They experienced difficulty putting away and taking out their prepaid IC cards and smartphones while walking, which took time or caused them to stop in crowds They could not handle prepaid IC cards with one hand while holding a cane with the other	10
Unable to pass through in line with the flow of people	They stopped walking because they could not move with the flow of people	7
Unable to use prepaid IC cards/smartphones at ticket gates	They could not use their IC cards or smartphones properly at the gate or did not know how to use them	6
Unable to handle tickets	They lost their ticket They forgot to take their ticket from the ticket gate	3
Passage through narrow spaces	They hit their paretic lower limb on the ticket gate	2
Unable to select the appropriate ticket gates	They attempted to enter a ticket gate that was impassable (e.g., opposite direction, closed)	2

**Selecting departure time and platform**		**202**

Unable to select platforms	They took a long time or needed assistance in selecting the platform of the train they wanted to board	28
Unable to check timetable/information display signs	They took a long time or required assistance to understand timetables and information boards due to aphasia or cognitive dysfunction They failed to see timetables or information boards owing to a lack of attention (including the effects of attention disorders, such as unilateral spatial neglect)	28
Unable to select a train	They tried to board trains that did not stop at the station they wanted to get off at For example, trying to board an express train when they should have boarded a local train or trying to board a train on a different line	8

**Getting on and off trains**		**203**

Losing balance	When stepping over the gap between the train and the platform, they lost their balance because they could not control their centre of gravity, or their feet were caught in the gap between the platform and the train	16
Improper movement procedures	They stepped out in the incorrect order (e.g., getting on from the paretic side first when one should get on from the non-paretic side) They got on and off without using a cane or handrail	14
Unable to get on/off in a short time	They could not move smoothly due to impatience and lack of attention They took time to switch canes to grip the handrail near the door of the train	9

**Getting on and off trains (continued)**		**203**

Unable to get on/off with the flow of people	They could not get on/off with the flow of people and consequently stopped moving, hindering others	9
Unable to select a station to get off at after getting on the train	They did not get off the train even when the train arrived at the station where they planned to get off They tried to get off at a different station	8
Difficulty going up and down steps	Owing to the large height difference between the platform and the train, they could not smoothly get on/off the train while walking, and consequently stopped moving	2

**Movement in trains**		**200**

Losing balance	They lost their balance when moving in a train, standing or sitting in their seat, or maintaining a standing position using a strap	9
Danger while moving when the train is running	They were unstable in their movements as they stood up before the train stopped	7
Danger while standing and sitting on a seat	They needed verbal assistance in timing their movements when getting up from their seats and sitting down, or physical assistance because their movements were unstable	5
Unable to select a suitable mobility method	They could not select a method that suited their mobility, such as walking without a cane or handrail, which made their movement unstable	2

**Selecting departure time and bus stop**		**185**

Unable to select a bus stop	They could not find the bus station or select the bus stop from which the bus they wanted to board would depart	11
Unable to check timetable/information display signs	They could not understand the departure time of buses as they were unable to check the timetables and confirm the time	11

**Getting on and off buses**		**180**

Losing balance	When stepping across the space between the bus and the bus stop, they lost control of their centre of gravity and their balance	23
Improper movement procedures	They stepped in the incorrect order (e.g., getting on from the paretic side first when one should get on from the non-paretic side) They got on and off without using a cane or handrail They tried to get on from inappropriately far away They were confused in the sequence of actions related to handling the cane or IC card (e.g., switching the cane from one hand to the other)	13
Unable to go up/down high and distant steps	Owing to the large height difference and distance between the bus and the bus stop, they had difficulty getting on/off the bus by themselves and needed physical assistance	6
Unable to get on/off in a short time	They could not move smoothly owing to impatience or lack of attention	4
Unable to get on/off with the flow of people	They could not get on/off with the flow of people and consequently stopped moving, obstructing others	3
Unable to go up/down steps carrying a cane/IC card	They could not use handrails or a cane to get up and down steps while holding an IC card	3

**Movement in buses**		**187**

Losing balance	They lost their balance when moving on the bus or standing by holding onto the strap	11
Danger owing to moving while the bus is running	They moved when the bus was running, which made their movements unstable	4
Difficulty standing up and sitting on the seat	Immediately after they boarded the bus, they could not decide which seat to take, and it took a long time for them to sit down They became unsteady when standing up from their seats	4

**Paying fares**		**190**

Unable to understand the payment procedure	They were unsure how to pay by touching a device or how to recharge their IC card or smartphone They were unsure how to exchange money They could not pay quickly and in accordance with the flow of people	12
Difficulty in preparing cash/IC card	They took a long time to retrieve cash (wallet), IC card, or smartphone because they had not prepared in advance for payment	7
Not understanding fares	They did not check the fare chart or did not know how to read it	3
Unable to handle tickets	They forgot to take their ticket when they boarded They lost their ticket	2

IC: Integrated Circuit.

The number listed on the same line as the task name indicates the number of participants who performed the task. The number listed on the same line as the problem indicates the number of participants for whom each classified problem was reported.

## DISCUSSION

This present study retrospectively analysed patient records of public transportation use practice on the PTAF during their admission to a rehabilitation hospital to determine the percentage of stroke patients who could perform each task independently and associated performance problems of tasks involved in using public transportation. A significant number of patients were unable to perform tasks independently and faced various problems in performing them. The task with the lowest percentage of independent participants was “Walking in crowds”, with 61.9% of participants performing it independently.

To the best of our knowledge, this study is the first to comprehensively and objectively evaluate the actual performance of post-stroke patients in using public transportation by using a standardized assessment tool. A previous study identified six tasks related to public transportation use and reported that individuals post-stroke most frequently encountered difficulty with “Getting on and off” both trains and buses (8). While that study provided valuable insights by identifying tasks for targeted interventions, the limited number of identified tasks did not capture the full range of tasks required for using public transportation. Moreover, as the previous study relied on self-report data, reliability issues, such as inconsistent criteria for assessing difficulty, cannot be ruled out. Conversely, our study employed the validated and reliable PTAF to determine the proportion of independent patients and performance problems across a wide array of tasks. The results provide more robust insights into the tasks that should be adequately assessed and practised in rehabilitation.

The percentage of participants who could independently perform all course items ranged from 15.4–41.9%. Over half of post-stroke patients who were expected to use public transportation had a limited ability to do so, which was consistent with prior studies (4, 11). Thus, post-stroke patients need additional support to use public transportation, including trains and buses. Improving the social infrastructure related to transportation, enhancing the healthcare system for support and practice, and developing rehabilitation intervention strategies that promote independence are necessary to eliminate restrictions on post-stroke patients’ social participation.

The low percentage of independent patients for “Walking in crowds” can be explained by slow gait speed and difficulties with multitasking, which are common among post-stroke patients. When walking in a crowd, one must pay attention to one’s surroundings so as not to get in the way of others. However, after a stroke, walking while paying attention to multiple tasks is critical (37, 38), and excessive auditory and visual stimuli while walking can make individuals unstable (39). Thus, the study participants experienced difficulties walking to their destination (or when looking for their destination) while continuing to pay attention to their surroundings. Additionally, stroke patients have a reduced walking speed (40), and they face challenges when walking in crowds, as they need to walk at a speed that matches the flow of the crowd. Another reason for challenges related to “Walking in crowds” could be the difficulty of practising in a hospital prior to a real-world setting. “Walking in crowds” is significantly influenced by external factors, such as the flow of the surrounding crowd and congestion; thus, it requires adapting to a continuously changing environment. Therefore, creating an environment that resembles the real-world setting for practising in the hospital is challenging and could hinder the adequate evaluation and practice of skills before actually using public transportation.

“Selecting departure time and platform” was another task with few independent patients. This could be because the participants were unfamiliar with the station they used in the practice, which was relatively large. Practising using public transportation in our study did not involve the trains and buses that the participants typically used but rather the courses prescribed by the hospital. Therefore, participants may have been unfamiliar with the station layout and train lines used in the practice. Additionally, the two stations used in the practice had multiple platforms, while the trains arriving and departing from these stations had several patterns depending on the number of stops (e.g., rapid train, express train) and destinations. Therefore, participants had to choose the correct trains and platforms in an unfamiliar environment, potentially making it difficult to understand information such as departure times and their location in the station. This finding is supported by a previous study that reported behavioural patterns in which post-stroke individuals avoided unfamiliar routes due to difficulties related to understanding bus schedules and the complexities of bus terminals (6). Note that after participants are discharged from hospital, it is not always necessary for them to check timetables and information signs at stations they are familiar with or at smaller stations. Therefore, more participants may be able to perform this task independently. However, by assessing performance at a station unfamiliar to the participants, the present study provides insights into the problems that post-stroke individuals may face when going to a station for the first time.

“Getting on and off trains” and “Getting on and off buses” were also tasks with few independent patients, consistent with an earlier community-based study (8). As there are often distance and height differences between station platforms and trains and between bus stops and buses, getting on and off them requires the ability to maintain balance. However, post-stroke patients often experience balance problems due to motor paralysis and other impairments (41, 42), making it difficult to maintain balance while taking large steps. Therefore, performing this task was physically demanding (6, 9, 43) and could have increased the risk of loss of balance. Further, owing to various impairments, post-stroke patients often take a long time to perform certain movements and face challenges when moving with the flow of people within the limited timeframe between when a train or bus arrives and when it departs (6, 9).

Common performance problems were identified for several tasks, including balance problems, fatigue, and improper procedures for performing the task. Balance problems (44) and fatigue (45) are common symptoms after stroke, and the results of the present study reveal that these symptoms directly affect the performance of some tasks related to the use of public transportation. Improper procedures in performing the task may suggest that cognitive impairment, one of the most common symptoms after stroke (46), prevented the participants from understanding the correct sequence of tasks and paying attention to this sequence when performing the task. Previous studies have reported that the presence of these symptoms (balance problems, fatigue, and cognitive impairment) is associated with low levels of independence in instrumental activities of daily living, including using transportation (47–49), thereby supporting the present study’s findings. These findings also suggest the need to adequately evaluate these symptoms in patients and their impact on task performance when using public transportation. Especially following a stroke, it is important to evaluate patients’ ability to learn procedures to perform tasks, as it can be difficult to acquire the skills to perform cognitively demanding tasks, such as tasks that require different and more complex procedures than those prior to the illness (50, 51). If the patient is unable to learn new procedures after repeated practice, continued practice until independence may not be very effective and compensatory measures, such as using different modes of transportation (18), may be recommended. Additionally, stroke patients also have a tendency to decrease their independence in activities of daily living when the environment changes from hospital to home (i.e., discharge from hospital to home) (52). Similarly in the use of public transportation, skills acquired during inpatient rehabilitation may not be utilized in a different setting following their discharge from a hospital. As such, there is a need to assess whether the skills learned are transferred for use in transportation in the community setting, and to consider how to follow up after discharge from a hospital.

One limitation of the present study was the use of a single rehabilitation hospital in Japan. Our results may have been influenced by the characteristics of the area’s public transportation system where the facility is located and by the characteristics of the patients admitted. Therefore, while the study results could be generalized to similar settings, it may not be possible to generalize them to different regions or countries with significantly different settings, including rural areas where the use of public transportation is particularly important for the social participation of people with stroke (13).

In conclusion, the present study identified the percentage of post-stroke patients able to independently perform 14 tasks related to the use of public transportation, along with problems that were encountered. A significant number of patients were unable to perform tasks independently and faced various problems in performing them. These issues should be addressed in the rehabilitation process to enable stroke patients to use public transportation once again.

## Supplementary Material

INVESTIGATION OF INDEPENDENCE IN THE TASKS INVOLVING THE USE OF PUBLIC TRANSPORTATION IN PATIENTS WITH SUBACUTE STROKE

## References

[1] Berger S, McAteer J, Schreier K, Kaldenberg J. Occupational therapy interventions to improve leisure and social participation for older adults with low vision: a systematic review. Am J Occup Ther 2013; 67: 303–311. 10.5014/ajot.2013.005447 23597688

[2] Obembe AO, Eng JJ. Rehabilitation interventions for improving social participation after stroke: a systematic review and meta-analysis. Neurorehabil Neural Repair 2016; 30: 384–392. 10.1177/1545968315597072 26223681 PMC4868548

[3] Chou CY. Determinants of the health-related quality of life for stroke survivors. J Stroke Cerebrovasc Dis 2015; 24: 655–662. 10.1016/j.jstrokecerebrovasdis.2014.10.022 25576350

[4] Wendel K, Ståhl A, Risberg J, Pessah-Rasmussen H, Iwarsson S. Post-stroke functional limitations and changes in use of mode of transportation. Scand J Occup Ther 2010; 17: 162–174. 10.3109/11038120902964450 19452360

[5] Finestone HM, Marshall SC, Rozenberg D, Moussa RC, Hunt L, Greene-Finestone LS. Differences between poststroke drivers and nondrivers: demographic characteristics, medical status, and transportation use. Am J Phys Med Rehabil 2009; 88: 904–923. 10.1097/PHM.0b013e3181aa001e 19487920

[6] Risser R, Iwarsson S, Ståhl A. How do people with cognitive functional limitations post-stroke manage the use of buses in local public transportation? Transp Res Part 2012; 15: 111–118. 10.1016/j.trf.2011.11.010

[7] Chen HF, Wu CY, Lin KC, Chen CL, Huang PC, Hsieh CJ, et al. Rasch validation of a combined measure of basic and extended daily life functioning after stroke. Neurorehabil Neural Repair 2013; 27: 125–132. 10.1177/1545968312457828 22941671

[8] Persson HC, Selander H. Transportation mobility 5 years after stroke in an urban setting. Top Stroke Rehabil 2018; 25: 180–185. 10.1080/10749357.2017.1419619 29334331

[9] Rosenkvist J, Risser R, Iwarsson S, Wendel K. The challenge of using public transportation: descriptions by people with cognitive functional limitations. J Transp Land Use 2009; 2: 65–80. 10.5198/jtlu.v2i1.97

[10] Asplund K, Wallin S, Jonsson F. Use of public transportation by stroke survivors with persistent disability. Scand J Disabil Res 2012; 14: 289–299. 10.1080/15017419.2011.640408

[11] Logan PA, Gladman JRF, Radford KA. The use of transportation by stroke patients. Br J Occup Ther 2001; 64: 261–264. 10.1177/030802260106400510

[12] Lord SE, McPherson K, McNaughton HK, Rochester L, Weatherall M. Community ambulation after stroke: how important and obtainable is it and what measures appear predictive? Arch Phys Med Rehabil 2004; 85: 234–239. 10.1016/j.apmr.2003.05.002 14966707

[13] Chavda K, Prakash V. Transportation use limitations and its association with social participation among patients with stroke living in rural India. Disabil Rehabil 2024; 46: 3980–3984. 10.1080/09638288.2023.2260740 37728331

[14] Bezyak JL, Sabella S, Hammel J, McDonald K, Jones RA, Barton D. Community participation and public transportation barriers experienced by people with disabilities. Disabil Rehabil 2020; 42: 3275–3283. 10.1080/09638288.2019.1590469 30991852

[15] Hammel J, Jones R, Gossett A, Morgan E. Examining barriers and supports to community living and participation after a stroke from a participatory action research approach. Top Stroke Rehabil 2006; 13: 43–58. 10.1310/5X2G-V1Y1-TBK7-Q27E 16987791

[16] Rimmer JH, Wang E, Smith D. Barriers associated with exercise and community access for individuals with stroke. J Rehabil Res Dev 2008; 45: 315–322. 10.1682/JRRD.2007.02.0042 18566948

[17] Norlander A, Iwarsson S, Jönsson A-C, Lindgren A, Lexell EM. Participation in social and leisure activities while re-constructing the self: understanding strategies used by stroke survivors from a long-term perspective. Disabil Rehabil 2022; 44: 4284–4292. 10.1080/09638288.2021.1900418 33779458

[18] Ståhl A, Månsson Lexell E. Facilitators for travelling with local public transportation among people with mild cognitive limitations after stroke. Scand J Occup Ther 2018; 25: 108–118. 10.1080/11038128.2017.1280533 28118764

[19] White JH, Miller B, Magin P, Attia J, Sturm J, Pollack M. Access and participation in the community: a prospective qualitative study of driving post-stroke. Disabil Rehabil 2012; 34: 831–838. 10.3109/09638288.2011.623754 22035162

[20] Ushizawa K, Otaka Y, Kitamura S, Inoue S, Sakata S, Kondo K, et al. Development of an assessment form for the performance of public transportation use in individuals with stroke. Disabil Rehabil 2023; 45: 2336–2345. 10.1080/09638288.2022.2089919 35764527

[21] Kitamura S, Otaka Y, Ushizawa K, Inoue S, Sakata S, Kondo K, et al. Reliability and validity of the public transportation use assessment form for individuals after stroke. Disabil Rehabil 2023; 45: 2346–2353. 10.1080/09638288.2022.2133175 36239400

[22] Logan PA, Gladman JRF, Avery A, Walker MF, Dyas J, Groom L. Randomised controlled trial of an occupational therapy intervention to increase outdoor mobility after stroke. BMJ 2004; 329: 1372–1374. 10.1136/bmj.38264.679560.8F 15564229 PMC535450

[23] Logan PA, Armstrong S, Avery TJ, Barer D, Barton GR, Darby J, et al. Rehabilitation aimed at improving outdoor mobility for people after stroke: a multicentre randomized controlled study (the Getting Out of the House Study). Health Technol Assess (Rockv) 2014; 18: vii–viii, 1–113. 10.3310/hta18290 PMC4781056 24806825

[24] Ogawa M, Sawada T, Ishizuki C, Okahashi S, Futaki T. Reasons for the lack of practice using public transportation at sub-acute rehabilitation hospitals in a Japanese urban community. Asian J Occup Ther 2016; 12: 67–74. 10.11596/asiajot.12.67

[25] Logan PA, Dyas J, Gladman JR. Using an interview study of transportation use by people who have had a stroke to inform rehabilitation. Clin Rehabil 2004; 18: 703–708. 10.1191/0269215504cr742oa 15473122

[26] Miyai I, Sonoda S, Nagai S, Takayama Y, Inoue Y, Kakehi A, et al. Results of new policies for inpatient rehabilitation coverage in Japan. Neurorehabil Neural Repair 2011; 25: 540–547. 10.1177/1545968311402696 21451116

[27] Chiba Prefectural Government. [Report on the Chiba prefecture monthly population survey]. 2017 [cited 2024 Aug 26]. Japanese. Available from: https://www.pref.chiba.lg.jp/toukei/toukeidata/joujuu/nenpou/2017/index.html

[28] Ministry of Land, Infrastructure, Transportation and Tourism: Kento Regional Development Bureau. [Result of a survey of Tokyo metropolitan area personal trip] [cited 2024 Aug 26]. [in Japanese]. Available from: https://www.tokyo-pt.jp/static/hp/file/press/1127press.pdf

[29] East Japan Railway Company [homepage on the Internet] [cited 2024 Aug 26]. Available from: https://www.jreast.co.jp/e/

[30] Keisei Electric Railway [homepage on the Internet] [cited 2024 Aug 26]. Available from: https://www.keisei.co.jp/

[31] Sugishita M, Hemmi I, Takeuchi T. Reexamination of the validity and reliability of the Japanese version of the Mini-Mental State Examination (MMSE-J). Jpn J Cogn Neurosci 2016; 18: 168–183 [in Japanese].

[32] Folstein MF, Folstein ES, McHugh RP. “Mini-mental state”: a practical method for grading the cognitive state of patients for the clinician. J Psychiatr Res 1975; 12(3): 189–198. 10.1016/0022-3956(75)90026-6 1202204

[33] Chino N, Sonoda S, Domen K, Saito E, Kimura A. Stroke Impairment Assessment Set (SIAS): a new evaluation instrument for stroke patients. Jpn J Rehabil Med 1994; 31: 119–125. 10.2490/jjrm1963.31.119

[34] Chino N, Sonoda S, Domen K, Saito E, Kimura A. Stroke Impairment Assessment Set (SIAS). In: Functional evaluation of stroke patients. Tokyo: Springer-Verlag Tokyo, 1996, p. 19–31. 10.1007/978-4-431-68461-9_3

[35] Data Management Service of the Uniform Data System for Medical Rehabilitation, Center for Functional Assessment Research. Guide for use of the uniform data set for medical rehabilitation, including the Functional Independence Measure (FIM), Version. Buffalo, NY: State University of New York Press, 1990.

[36] Keith RA, Granger C V, Hamilton BB, Sherwin FS. The functional independence measure: a new tool for rehabilitation. Adv Clin Rehabil 1987; 1: 6–18. 3503663

[37] Yang YR, Chen YC, Lee CS, Cheng SJ, Wang RY. Dual-task-related gait changes in individuals with stroke. Gait Posture 2007; 25: 185–190. 10.1016/j.gaitpost.2006.03.007 16650766

[38] Smulders K, van Swigchem R, de Swart BJM, Geurts ACH, Weerdesteyn V. Community-dwelling people with chronic stroke need disproportionate attention while walking and negotiating obstacles. Gait Posture 2012; 36: 127–132. 10.1016/j.gaitpost.2012.02.002 22418584

[39] Törnbom K, Sunnerhagen KS, Danielsson A. Perceptions of physical activity and walking in an early stage after stroke or acquired brain injury. PLoS One 2017; 12: e0173463. 10.1371/journal.pone.0173463 28273158 PMC5342245

[40] Dean CM, Richards CL, Malouin F. Walking speed over 10 metres overestimates locomotor capacity after stroke. Clin Rehabil 2001; 15: 415–421. 10.1191/026921501678310216 11518442

[41] Lewek MD, Bradley CE, Wutzke CJ, Zinder SM. The relationship between spatiotemporal gait asymmetry and balance in individuals with chronic stroke. J Appl Biomech 2014; 30: 31–36. 10.1123/jab.2012-02088 23677889

[42] Tyson SF, Hanley M, Chillala J, Selley A, Tallis RC. Balance disability after stroke. Phys Ther 2006; 86: 30–38. 10.1093/ptj/86.1.30 16386060

[43] Barnsley L, McCluskey A, Middleton S. What people say about travelling outdoors after their stroke: a qualitative study. Aust Occup Ther J 2012; 59: 71–78. 10.1111/j.1440-1630.2011.00935.xx 22272885

[44] Khan F, Chevidikunnan MF. Prevalence of balance impairment and factors associated with balance among patients with stroke: a cross-sectional retrospective case control study. Healthcare 2021; 9: 320. 10.3390/healthcare9030320. 33805643 PMC7998930

[45] Cumming TB, Packer M, Kramer SF, English C. The prevalence of fatigue after stroke: a systematic review and meta-analysis. Int J Stroke 2016; 11: 968–977. 10.1177/1747493016669861. 27703065

[46] Sexton E, McLoughlin A, Williams DJ, Merriman NA, Donnelly N, Rohde D, et al. Systematic review and meta-analysis of the prevalence of cognitive impairment no dementia in the first year post-stroke. Eur Stroke J 2019; 4: 160–171. 10.1177/2396987318825484. 31259264 PMC6591758

[47] Rand D. Mobility, balance and balance confidence: correlations with daily living of individuals with and without mild proprioception deficits post-stroke. NeuroRehabilitation 2018; 43: 219–226. 10.3233/NRE-172398 30175987

[48] Port IGL van de, Kwakkel G, Schepers VPM, Heinemans CTI, Lindeman E. Is fatigue an independent factor associated with activities of daily living, instrumental activities of daily living and health-related quality of life in chronic stroke? Cerebrovasc Dis 2007; 23: 40–45. 10.1159/000095757 16968985

[49] Rohde D, Gaynor E, Large M, Mellon L, Hall P, Brewer L, et al. The impact of cognitive impairment on poststroke outcomes: a 5-year follow-up. J Geriatr Psychiatry Neurol 2019; 32: 275–281. 10.1177/0891988719853044 31167593

[50] Kitamura S, Otaka Y, Murayama Y, Ushizawa K, Narita Y, Nakatsukasa N, et al. Difficulty of the subtasks comprising bed–wheelchair transfer in patients with subacute strokes: a cohort study. J Stroke Cerebrovasc Dis 2022; 31: 106740. 10.1016/j.jstrokecerebrovasdis.2022.106740 36054975

[51] Kitamura S, Otaka Y, Murayama Y, Ushizawa K, Narita Y, Nakatsukasa N, et al. Differences in the difficulty of subtasks comprising the toileting task among patients with subacute stroke: a cohort study. J Stroke Cerebrovasc Dis 2023; 32: 107030. 10.1016/j.jstrokecerebrovasdis.2023.107030 36709731

[52] Somerville E, Blenden G, Kretzer D, Holden B, Bollinger RM, Krauss MJ, et al. Differences in daily activity performance between inpatient rehabilitation facility and home among stroke survivors. Neurorehabil Neural Repair 2024; 38: 403–412. 10.1177/15459683241246266 38602200 PMC11100317

