# Supplementary material for: INVESTIGATION OF INDEPENDENCE IN THE TASKS INVOLVING THE USE OF PUBLIC TRANSPORTATION IN PATIENTS WITH SUBACUTE STROKE
Source: J Rehabil Med. 2025 Mar 12;57:42025. doi: 10.2340/jrm.v57.42025 (PMC13064439; doi:10.2340/jrm.v57.42025)

Fig. S1. Public transportation use assessment form

Patient name: (years old)	Diagnosis (affected side):		Admission date:	Assessment date:	Expected discharge date:
Accompanying staff name:	Accompanying family: Y/N	Accompanying family name:	Public transportation used: 1 (train) / 2 (bus) / 3 (train and bus)		
Training purpose:					

For the evaluation result, enter 3 (independent), 2 (requires supervision or verbal assistance), 1 (requires assistance), N (not applicable). For 2 or 1, describe the specific problems and countermeasures.
The total score was calculated by dividing the total score by the number of items (excluding the items marked as not applicable [N]).

Category	Assessment item	Examples	Assessment	Problems	Countermeasures
Plan for going out	Creating and understanding plans	Creating plans as needed, assessment understanding (e.g., route, time required, cost)			
Mobility	Movement over long time	Continuous walking, walking with breaks			
	Walking in crowds	Consideration of surroundings and flow of people (movement in line with flow, movement avoiding flow)			
	Using outdoor stairs				
	Using escalators				
Using trains	Operating ticket machines	Checking fare, buying ticket, charging IC card, asking station staff			
	Passing through ticket gates	Passing through smoothly, readying and inserting ticket, readying and tapping IC card, asking station staff			
	Selecting departure time and platform	Checking timetables and information displays, asking others			
	Getting on and off trains	Going up and down steps safely, consideration of flow of people (getting on and off in line with flow, getting on and off avoiding flow)			
	Movement in trains	Moving safely, sitting on and standing from seats, maintaining standing position (using handrails or straps)			
Using buses	Selecting departure time and bus stop	Checking timetables and information displays, asking others			
	Getting on and off buses	Going up and down steps safely, consideration of flow of people (getting on and off in line with flow, getting on and off avoiding flow)			
	Movement in buses	Moving safely, sitting on and standing from seats, maintaining standing position (using handrails or straps)			
	Paying fares	Checking fare, taking ticket, readying and inserting cash, readying, charging, and tapping IC card, asking driver			
Other	Other problems observed				
Summary					Total score:

Fig. S2. Courses of practice for public transportation use

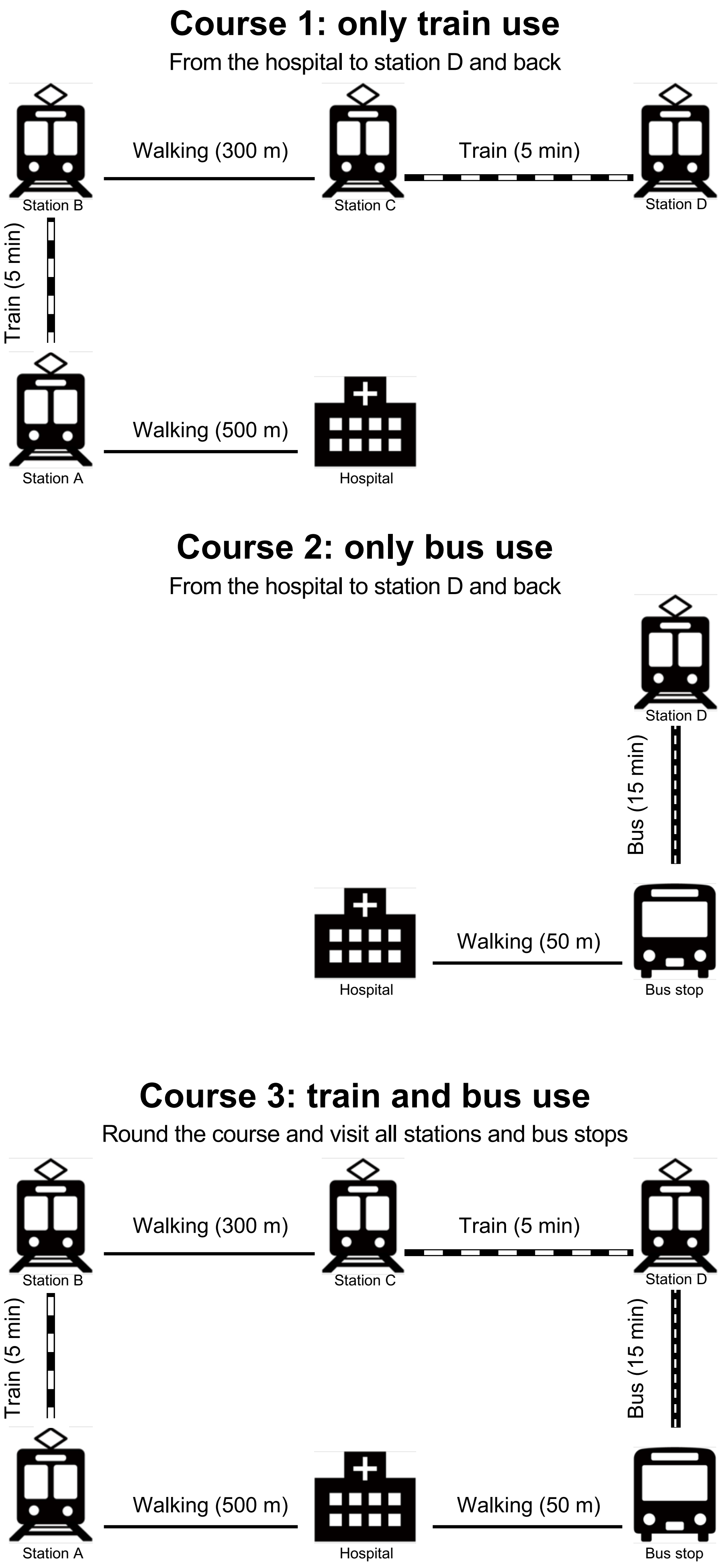

Supplement: INVESTIGATION OF INDEPENDENCE IN THE TASKS INVOLVING THE USE OF PUBLIC TRANSPORTATION IN PATIENTS WITH SUBACUTE STROKE [file JRM-57-42025-s1.pdf]
